# Low Intensity Noise Exposure Enhanced Auditory Loudness and Temporal Processing by Increasing Excitability of DCN

**DOI:** 10.1155/2022/6463355

**Published:** 2022-11-21

**Authors:** Lin Shi, Katie Palmer, Haolin Wang, Matthew A. Xu-Friedman, Wei Sun

**Affiliations:** ^1^Department of Otorhinolaryngology, The First Hospital of Dalian Medical University, Dalian, China; ^2^Department of Communicative Disorders and Sciences, Center for Hearing and Deafness, State University of New York at Buffalo, Buffalo, NY, USA; ^3^Department of General Surgery, The First Hospital of Dalian Medical University, Dalian, China; ^4^Department of Biological Sciences, State University of New York at Buffalo, Buffalo, NY, USA

## Abstract

Sound stimulation is generally used for tinnitus and hyperacusis treatment. Recent studies found that long-term noise exposure can change synaptic and firing properties in the central auditory system, which will be detected by the acoustic startle reflex. However, the perceptual consequences of long-term low-intensity sound exposure are indistinct. This study will detect the effects of moderate-level noise exposure (83 dB SPL) on auditory loudness, and temporal processing was evaluated using CBA/CaJ mice. C-Fos staining was used to detect neural activity changes in the central auditory pathway. With two weeks of 83 dB SPL noise exposure (8 hours per day), no persistent threshold shift of the auditory brainstem response (ABR) was identified. On the other hand, noise exposure enhanced the acoustic startle response (ASR) and gap-induced prepulse inhibition significantly (gap-PPI). Low-level noise exposure, according to the findings, can alter temporal acuity. Noise exposure increased the number of c-Fos labeled neurons in the dorsal cochlear nucleus (DCN) and caudal pontine reticular nucleus (PnC) but not at a higher level in the central auditory nuclei. Our results suggested that noise stimulation can change acoustical temporal processing presumably by increasing the excitability of auditory brainstem neurons.

## 1. Introduction

Noise exposure is an ordinary origin of sensorineural hearing loss, and it is also related to tinnitus and hyperacusis [1]. As observed with high noise, low noise exposure has been reported to affect brain processing of acoustic stimuli, intensity, and frequency domains in several animal investigations. For example, animal studies have found that cochlear lesions can lead to substantial plasticity changes in the frequency of the central auditory system near the hearing loss area [2]. These plasticity changes, including reduction of central inhibition, firing rate increase, and expansion of the tonotopic map of the auditory cortex (AC) may cause aberrant sound perception related to tinnitus and hyperacusis [3, 4]. Studies also found that sound treatment right after high-level noise exposure, which may compensate for reduced peripheral inputs, may prevent hearing-loss-induced tinnitus and hyperacusis [3]. Sound stimulation has also been used as a treatment to reduce sound intolerance in tinnitus patients with hyperacusis [5]. Although there were plentiful studies investigating the perceptual consequences of long-term noise exposure at moderate levels, for example, taking Chen's study as an example, young rats were found to be more susceptible in an environment with long-term noise, which affects cortical neuron perception of sound levels [6]. Besides, Cheng et al. discovered that when animals are exposed to short-term moderate noise, sound level processing in auditory midbrain neurons is impaired in a frequency-specific manner [7], and they also revealed that rats bred in noisy environments had lower temporal processing acuity than adults, possibly because of an ion channel defect [8], small silent gap (1-100 ms) buried in background noise causes gap-induced prepulse inhibition (gap-PPI). Few studies have focused on alterations in the central nucleus when low-intensity noise does not induce a shift in hearing threshold.

The acoustic startle response (ASR), a reflexive defensive response to a sudden sound stimulus, has been used to evaluate sound loudness and temporal processing [9–11]. The ASR involves the auditory nerves and circuits in the auditory brainstems [12], and it can be modulated if an acoustic signal precedes the sound-induced ASR, called prepulse inhibition [13]. Prepulse inhibition can be used to evaluate sensorimotor gating deficits commonly found in schizophrenic patients [14]. Gap-induced prepulse inhibition (gap-PPI) is led by a short silent gap (1-100 ms) inserted in the noise and can be used to evaluate gap detection and temporal acuity [15]. As the measurement of ASR and prepulse inhibition does not require auditory training, it can be used as a convenient assessment of auditory processing [15].

Recently, a modified gap-PPI procedure using long gaps (50 ms) has been used to assess for tinnitus in animal models [16]. Surprisingly, animals given large amounts of salicylate, the active ingredient in aspirin that causes tinnitus in people did not develop tinnitus in animals which showed an augmented ASR despite a 30-40 dB hearing loss [11]. The unprotected animals also showed an evidential reduction of gap-PPI. Although there is still debate on using this method, many studies found that these tests can help to reveal the mechanisms underlying tinnitus and hyperacusis. To evaluate the degree of using moderate noise exposure on auditory loudness and temporal processing acuity, acoustic startle response and gap-PPI were measured in CBA-CaJ mice before and after being raised in a moderate noise environment. To examine the auditory nuclei involved in the perceptual modifications, the c-Fos gene in the central auditory system was used to evaluate stimulus-related local neuronal activation at the cellular level [17].

## 2. Materials and Methods

### 2.1. Experimental Animals

Twenty CBA/CaJ mice (male and female, 2 months old) reared at the University at Buffalo animal Facility were used in this study (Jackson Lab, Bar Harbor, ME). The mice were randomly divided into two groups: control group (*n* =  10) and noise group (*n* = 10). All tests were approved by the Institutional Animal Care and Use Committee (IACUC) at The State University of New York at Buffalo and followed the National Institutes of Health guidelines.

### 2.2. Auditory Brainstem Response (ABR) Recording

**Figure 1:**
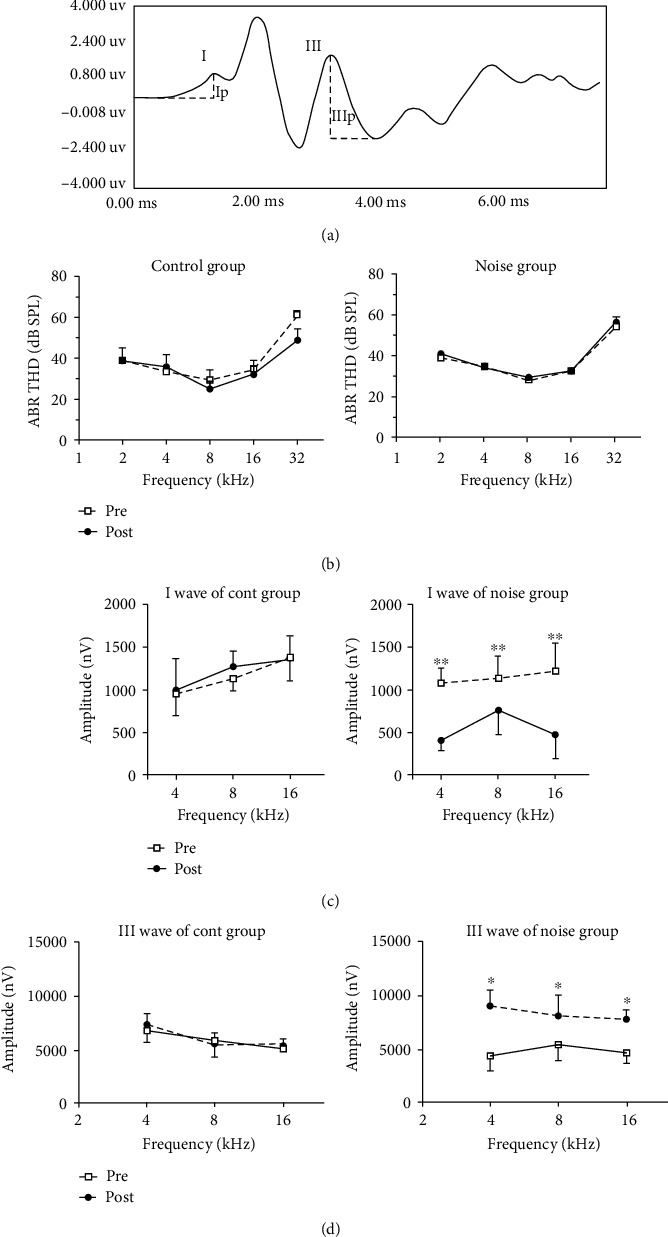
Auditory brainstem response (ABR) was measured in control and noise-exposed mice. The noise groups were exposed to moderate-level background noise (83 dB SPL) for 8 hrs each day for 2 weeks. (a) A typical ABR response at 90 dB SPL, with waves I and III marked. (b) The average ABR threshold in control and noise groups. There was no significant difference in the ABR thresholds in the noise group compared to controls. (c) The amplitudes of wave I at 90 dB SPL of both groups before and after noise exposure. Significant decrease in the amplitude of wave I was detected in the noise group (*p* < 0.05), especially at 16 kHz (*p* < 0.01). (d) The amplitudes of wave III at 90 dB SPL of both groups. Significant increase of wave III amplitude was detected in the noise group at 4, 8, and 16 kHz (*p* < 0.05).

After anesthesia with pyrazine (10 mg/kg) and ketamine (100 mg/kg), ABRs in both groups were measured using BioSigRP software and TDT System-3 (Tucker Davis Technology, FL, USA). Different needle electrodes are placed at the apex and opposite auricle of the ear to be measured as noninverted (+) input and ground wires to evaluate the ABR threshold. We used a silver ball electrode on the tympanic membrane of the stimulated ear as a reference input to get the steady wave response of ABR. Tone bursts at 2, 4, 8, 16, and 32 kHz (2 ms duration, 0.1 ms rise/fall time) were swept in levels from 10-90 dB SPL with a step of 5 dB SPL to detect the minimal intensity which elicited a response. The bandpass filter for ABR acquisition was set at 100-3000 Hz in BioSigRP software. As mentioned in previous study [18], the wave I amplitude was recorded as the peak to basement, and the amplitude of wave III was measured as the amplitude between the positive peak and the following negative peak (Figure 1(a)).

### 2.3. ASR and Gap-PPI Test

**Figure 2:**
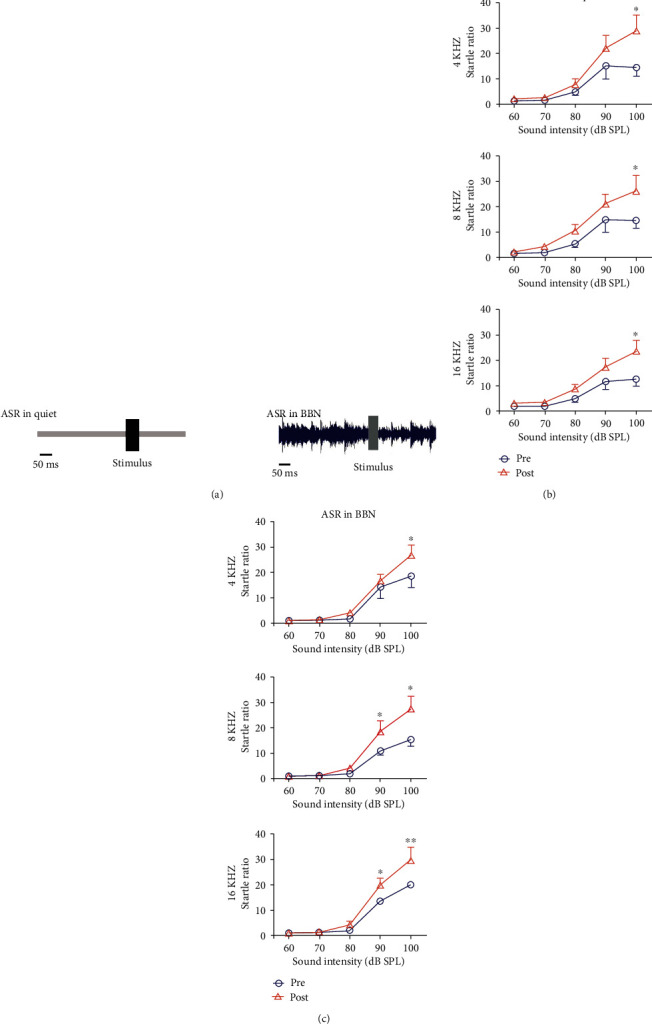
Behavioral assessment of animals by acoustic startle reflex. (a) Acoustic startle response was measured in a quiet background (left) and a broadband background noise (BBN) at 60 dB SPL (right). The startle sound was a narrow band noise burst centered at 4, 8, and 16 kHz (50 ms duration, 60-100 dB SPL in 10 dB step). (b) The startle response was measured before and after 2 weeks of noise exposure. Clearly an increase of the startle response was detected in the noise group (Two-way ANOVA, *p* < 0.05). (c) Startle amplitudes tested under BBN before and after 2 weeks of noise exposure. Significant increases can be detected after noise exposure. (two-way ANOVA test and Bonferroni posttest, ^∗^*p* < 0.05, ^∗∗^*p* < 0.01).

In our previous paper [9, 19], we established the approach for ASR and gap-PPI testing. Mice were fixed in a tiny mesh container (7 × 3 × 5) cm in size before testing to limit their activity. To minimize sound reflection, the container was put in a basement room with a sensitive piezoelectric sensor and each wall shielded with a one-inch-thick layer of solid foam. To improve the output of the piezo transducer, a low-pass filter (LPF-300, World Precision Instruments, Sarasota, FL, USA) was utilized, followed by an A/D converter on an RP2 real-time CPU (TDT). Sound stimulation was output by a speaker (FT28D) 28 cm above the rat heads. The ASR stimulation was done in a limited band centered at 4, 8, and 16 kHz to assess the association between the amplitude of startle and the intensity of sound stimulation. It has a noise burst (length 50 ms, rise/fall time 1 ms, bandwidth 2 kHz) that appears at random with a 60-100 dB SPL intensity (10 dB steps, 10 tests per condition). The Interval of Stimulus (ISI) fluctuates between 18 and 23 seconds at random. To avoid the habits associated with repeating stimuli, sound stimuli occur at random levels. White noise at 60 decibels SPL was utilized as a background sound to see if it affected the startle reflex. The magnitude of the startle was examined in response to the presence or absence of noise (Figure 2(a)).

**Figure 3:**
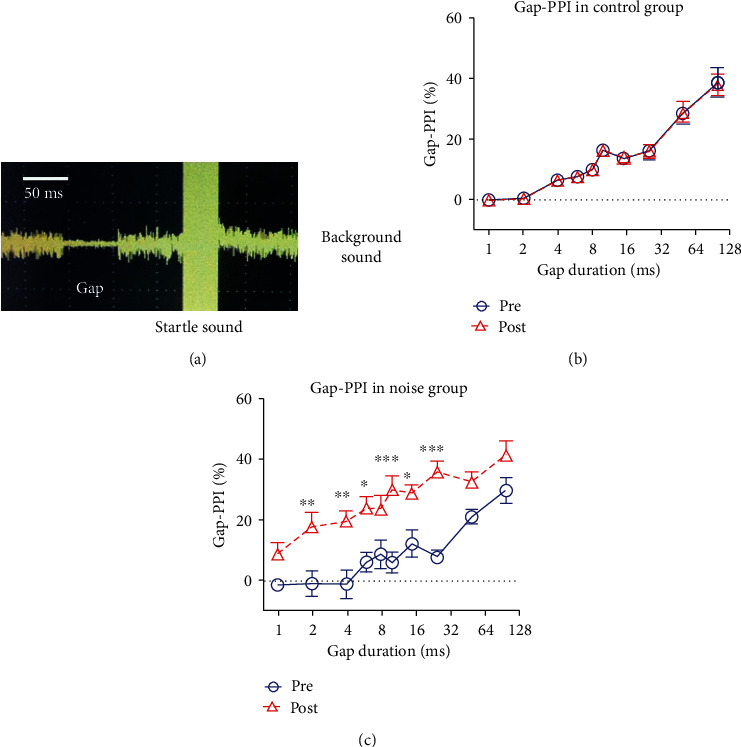
Measurement of gap-induced prepulse inhibition (gap-PPI) of the auditory startle response (ASR). (a) A diagram of noise used in the gap-PPI test. The gap duration was 1 to 100 ms, and the background noise was 70 dB SPL. (b, c) gap-PPI recorded in the control and noise groups. The gap PPi at 6, 8, 10, 15, and 25 ms of the noise group was significantly higher than the control group (*n* = 10, Two-way ANOVA, ^∗^*p* < 0.05).

We looked at the startle reaction to the presence or absence of a quiet gap in background wideband noise (1-20 kHz) at 70 dB SPL to find gap PPI. The silent gap is 60 milliseconds before the startle stimulus and lasts 1 to 100 milliseconds (1, 2, 4, 6, 8, 10, 15, 25, 50, and 100 milliseconds).  Figure 3(a) shows an example of an acoustic stimulation waveform taken by a digital oscilloscope. The startle amplitude was determined using a recording window that opened 180 milliseconds after the startle sound began. The following formula is used to compute the gap PPI:

(1) $$\begin{array}{c} \text{Gap}-\text{PPI}=\left(\frac{\text{AS}{\text{R}}_{\text{no}-\text{gap}}-{\text{ASR}}_{\text{gap}}\;}{{\text{ASR}}_{\text{no gap}}\;}\right)\times 100\%. \end{array}$$

All various sound treatments were blended for each test session, and the order of trials was doubly random between trials. Mice were tested for up to 2 hours each day to avoid fatigue, and each test type (startle with or without background sound, gap-PPI) was only done once per mouse. There was a 2-minute acclimatization period at each stage before the startle test, and all tests were performed in complete darkness.

### 2.4. Noise Exposure

The noise group was subjected to 83 dB SPL white noise for 8 hours each day for 14 days (10 pm to 6 am). A white noise generator (ACO Pacific 3025) produced the acoustic stimuli, which was delivered through a speaker (FT 28 D) placed 10 cm above the mice, exposing both ears. To guarantee steady noise circumstances, acoustic levels must be evaluated before and after each exposure. The control animals were raised in an environment with a background noise level of no more than 60 decibels SPL.

### 2.5. Immunohistochemistry

Following the behavioral experiments, mice were killed for c-Fos staining. Due to c-Fos expression being induced most effectively 2-3 hours after auditory stimulation, 20 mice were exposed to 80 dB SPL white noise for 1 hour before being put in a quiet anechoic cage for another hour. Following that, the mice were anesthetized with ketamine and xylazine, and PBS was injected into the ascending artery with buffer formaldehyde with 10% phosphate. For protection, the brain was settled in 10% formalin overnight and then frozen in PBS with 30% sucrose in a slide sectioner (HM 505 N) equipped with a freezing platform, freezing slices with a crown cut of 40 *μ*m. All slices were collected and kept at 4°C in PBS. Free floating slices from the dorsal cochlear nucleus (DCN) to AC were collected at 0.1 M PBS, and c-Fos immunohistochemical treatment was performed on the tissue slices.

Diaminobiphenylamine (DAB) and immunofluorescent agents were used to stain brain sections. The slices were hatched with 0.3 percent H2O2 and submerged in a blocking solution after being rinsed in PBS (pH 7.4) (0.1 percent bovine serum albumin), and then incubated for 24 hours at 4° in a 1: 500 diluted solution of mouse c-Fos monoclonal antibody (sc-166940, Santa Cruz Biotechnology, Inc.). Slices were washed with PBS before being incubated with a closed solution diluted 1 : 100 with anti-mouse IgG antibody (BA-2001, Vector Laboratories, Inc.) biotinylated at ambient temperature for 1.5-2 hours before reacting with ABC (Burlingame, CA) (Avidin-Biotin Complex in blocking solution, 1 : 50, PK-6100, Vector Laboratories, Inc. Burlingame, CA). Slices were incubated in a 0.05 percent solution of 3,3′-diaminobiphenylamine (DAB, D 5905, Sigma Chemical) for 5 minutes. The staining effect was shown with 0.001% H2O2. After washing the slices with PBS, they were attached to a gelled slide glass, dried, graded, dehydrated by alcohol, and then covered with the slide glass. A light microscope was used to examine the slides (Axioskop, Zeiss, Germany). For each slide, 2.5 × len and 40 × len were used to observe.

Brain sections were immunofluorescent labeled with blocking buffer solution (10% normal donkey serum, 0.3% Triton X-100, and 0.1% sodium azide) or 30 minutes at room temperature before mice conjugated to Alexa-594. Incubated overnight with a Fos monoclonal antibody (1 : 50, sc-166940, Santa Cruz) room and washed with 0.1 M PBS. After 20 minutes of incubation at room temperature with To-PRO-3 iodide antibody (1: 200, T 3605, Life technology, US), the slices were rinsed three times with 0.1 M PBS for 10 minutes each time. Slices were visually and quantitatively analyzed under a 40x magnifying glass using a chase laser confocal microscope (Zeiss LSM 510). The excitation wavelength was set to 555 nm, and the emission wavelength was 647 nm. A sequence scan was performed for each site, and the scan interval is 2.5 *μ*m.

### 2.6. C-Fos Counting

**Figure 4:**
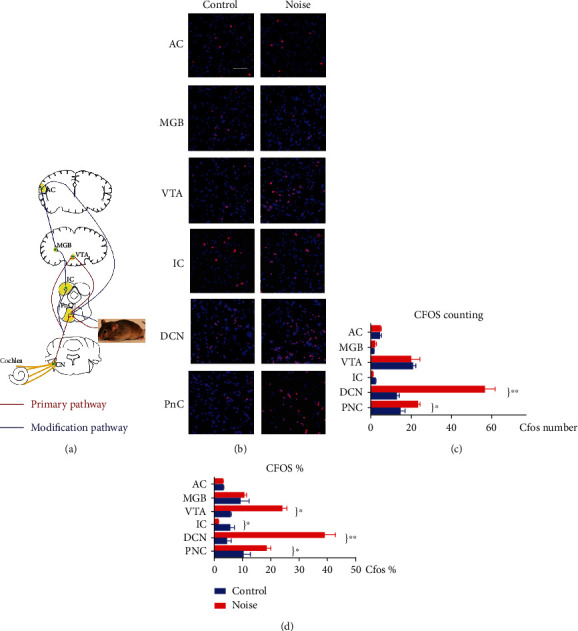
C-Fos expression in different sites of the auditory pathway. (a) Schematic of the primary ascending pathway and the descending modulatory pathway of acoustic startle response (ASR). (b) Immunofluorescence results of the caudal pontine reticular nucleus (PnC), the inferior colliculus (IC), dorsal cochlear nucleus (DCN), medial geniculate body (MGB), ventral tegmental area (VTA), and the auditory cortex (AC). (c) The number of c-Fos-positive cells in the noise-exposed group was significantly higher than the control group in the PnC and DCN. (d) The proportion of c-Fos staining in different parts of the auditory pathway. The c-Fos in the DCN, PnC and VTA of the noise group was significantly higher than the control group (^∗^*p* < 0.05).

Continuously scanned 2D images were used to calculate c-Fos positive cell nuclei. Observe slices from top to bottom using ZEN lite software 2012 (Carl Zeiss, Germany). To detect c-Fos, the photos were amplified in “zoomed top view.” We counted the number and percentage of c-Fos in each slice in our investigation and measured the auditory passage slices at every 25 m, including the DCN, the caudal pontine reticular nucleus (PnC), and the inferior colliculus (IC), the medial geniculate body (MGB), ventral tegmental area (VTA), and the AC (Figure 4(a)).

ImageJ software was used for cell counting of confocal images. A 40x magnification on the confocal microscope was used for counting cells in an area of ×160 *μ*m. If there is a positive marker (blue spot) of TO-PRO-3, it is considered as a cell. The red fluorescence point combined with the blue marker on each image was marked as c-Fos (+). ImageJ software (NIH, Bethesda, MD, USA) was used to calculate copositioning on a series of contiguous optical slices with 2.5 m increments along the slice's Z axis. Each animal had three slices of each brain area tallied, with each slice collecting five pictures. ImageJ software (NIH, Bethesda, MD, USA) was used on a series of contiguous optical slices to determine copositioning with an increment of 2.5 *μ*m along the Z axis of the slice. For each mouse, 3 slices of each brain region were counted, and each slice collected 5 images.

### 2.7. Statistical Analysis

GraphPad Prism (GraphPad Software, San Diego, CA, USA) was used for statistical analysis and graphing. Values are expressed as mean ± SEM. Two-factor ANOVA and Bonferroni posttest were used for statistical analysis.

## 3. Results

### 3.1. ABR Threshold Shift and Amplitudes of Wave I and Wave III

The ABR thresholds of CBA/CaJ mice in the noise group (*n* = 10) were determined before and 2-4 hours post noise exposure, right after the startle responses were tested. The averaged ABR thresholds (*n* = 10) before and after noise exposure were shown in Figure 1. There were few significant differences in the noise group before and after noise exposure (Two-way ANOVA test, *p* > 0.05). A control group of mice (*n* = 10), which were raised in a regular housing environment, were also tested on the same day to rule out the hearing loss affected by other causes. The control group also did not show any significant difference compared to the noise group (Two-way ANOVA test, *p* > 0.05, Figure 1(b)).

The amplitudes of ABRs (wave I and wave III) were also measured. Before noise exposure, the wave amplitudes of the noise group were 1081.5 ± 115.8 nV, 1137.4.5 ± 220.9 nV, and 1200.1 ± 253 nV at 4, 8 and 16 kHz, respectively. There was no momentous difference between the two groups. After noise exposure, the amplitude decreased to 407.1 ± 130.5 nV, 661.8 ± 153.2 nV, and 462 ± 130.1 nV at 4, 8, and 16 kHz, respectively, in the noise group. The difference between pre and postnoise exposure of the noise group was significant (Figure 1(c), two-way ANOVA). Bonferroni posttest showed significance at 4, 8, and 16 kHz, *p* < 0.01. By contrast, the amplitude of wave III in the noise group showed a clear increase after noise exposure (Two-way ANOVA, *p* < 0.01) and Bonferroni posttest showed significant at 4, 8, and 16 kHz (*p* < 0.01, Figure 1(d)).

### 3.2. ASR and Gap-PPI

To test how noise exposure affected sound loudness perception, the strength of the ASR was assessed before and after noise exposure. The results of ASR amplitude responses under the stimulus at 4, 8, and 16 kHz at different intensities are shown in Figure 2. Sound over 80 dB SPL elicited a clear startle response and the ASR amplitude increased significantly with sound intensity. The startle amplitude increased significantly after noise exposure at 4, 8, and 16 kHz (Two-way ANOVA test, the *F* (1,90) values were 19.54, 63.91, and 53.01 at 4, 8, and 16 kHz, respectively, *p* < 0.01; Bonferroni postest showed the difference at 100 dB for 4 kHz, at 80, 90, and 100 dB for 8 and 16 kHz, *p* < 0.05, Figure 2(b)). With a 60 dB SPL white noise, the startle responses were also used to test if a background would affect the startle amplitude. The ASR responses also showed a sharp increase after noise exposure in the presence of background noise (Figure 2(c), two-way ANOVA test, *F* (1,90) values were 17.08, 44.78, and 65.64 at 4, 8, and 16 kHz, respectively, *p* < 0.01); The Bonferroni posttest presents difference at 100 dB for 4 kHz, at 90 and 100 dB for 8, and 16 kHz, *p* < 0.0). Comparing the startle amplitudes measured with/without background noise, there was no statistically significant difference (Two-way ANOVA test, *p* > 0.05). There was no change happened in the control group measured over two weeks.

In the gap-PPI test, a silent gap with a background noise (70 dB SPL) greater than 6 ms significantly inhibited the startle response in control mice, and longer gaps induced an increase of gap-PPI (Figure 3(b)). After noise exposure, the mice showed a significant increase in gap-PPI. For 2 to 25 ms gaps, the average gap-PPI increased from 5.6% (0-12.2%) to 25.7% (*n* = 8, 17.8-35.9%) after noise exposure (Two-way ANOVA, *F* (1, 140) = 107.8, *p* < 0.001; Bonferroni posttest showed significance at 2, 4, 6, 10, 15, and 25 ms, Figure 3(c)). No significant change was detected in the control group before and after the experiment (Figure 3(b)).

### 3.3. C-Fos Staining and Counting

When viewed at high magnification, c-Fos-positive immunostaining was detected in cellular nuclei. The c-Fos-positive staining particles of DCN and PnC were significantly increased in the noise group (Figures 4(b) and 4(c)). The average numbers of positive neurons were 56.3 ± 5.4 in the DCN and 23 ± 1 in the PnC in the noise group and 12.5 ± 1.4 in the DCN and 14.3 ± 2.6 in the PnC in the control group. There were statistically significant differences between the two groups (Two-way ANOVA test, *F* (1,108) = 499, p<0.01). (Two-way ANOVA test, *F* (1,108) = 499, *p* < 0.01). According to Bonferroni posttest, it was found that DCN and PnC were the most clearly changed nuclei, while the IC, the VTA, the MGB, or the AC did not change (*p* > 0.05). Comparing the percentage of neurons that were c-Fos positive, we found that the difference between the two groups is large (Two-way ANOVA test, *F* (1,108) = 1083.7, *p* < 0.001). Posttest also showed a higher percentage in the VTA of the noise group animals (Figures 4(b) and 4(d), 5.60 ± 0.48% for the control group and 23.97 ± 1.75% for the noise group, *p* < 0.001). The IC pictured clearly a reduction in c-Fos-positive cells after noise exposure, 5.42 ± 1.72% for the control group and 1.43 ± 0.29% for the noise group (*p* < 0.05). There was no significant difference in MGB and AC between the two groups (*p* > 0.05).

**Figure 5:**
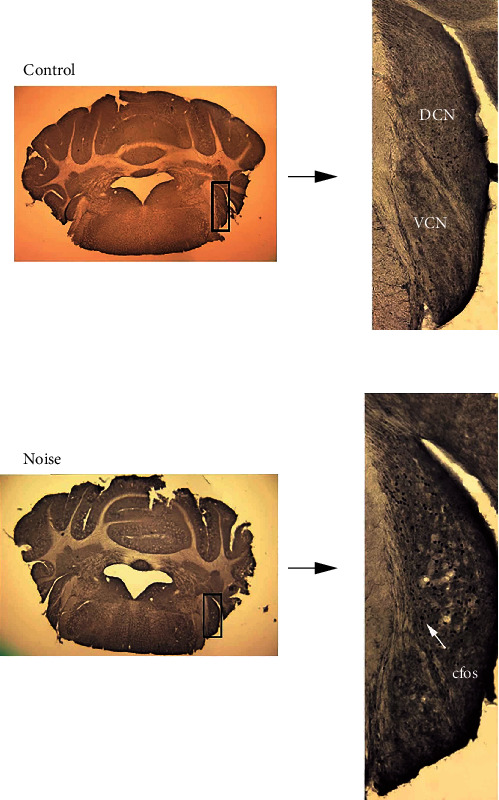
Comparison of c-Fos expression in the two groups of animals at the cochlea nucleus. The expression of c-Fos in the cochlea nucleus of the noise group was significantly higher than that in the control group. The expression site is mainly located in DCN. A circular black spot is c-Fos positive (↖).

With the antibody used in DAB staining, we observed a robust staining of c-Fos-positive neurons in the DCN of noise-exposed animals (Figure 5). After 14-day exposure, c-Fos-positive neurons occupied a larger nuclear area in DCN but were present at a low density in VCN in the noise group. And the expression of c-Fos in DCN in the noise group was more than that in the control group.

## 4. Discussion

One of the interesting findings of this research is that long-term moderate noise exposure (83 dB SPL for 2 weeks) caused a significant increase in the acoustic startle response of CBA/CaJ mice without impairing their hearing threshold. In addition, the noise-exposed mice showed increased inhibition to silent gaps, indicating an enlarged sensitivity in temporal processing [15]. We also found enhanced ABR wave III amplitudes and more c-Fos-positive neurons in the DCN and the PnC in the noise group, suggesting that the increase of auditory brainstem excitability may contribute to the auditory processing enhancement.

### 4.1. Low Intensity Noise Enhanced ASR and Neural Activity of the Primary Startle Reflex Pathway

Hypersensitivity to a loud sound is presumably caused by the increased response within the central auditory system and is a general situation in tinnitus and hyperacusis patients [20]. In previous studies, several experiments have demonstrated that when exposed to certain noises, among them, moderate hearing loss can also induce an increase in the amplitude of suprathreshold sound startle [21, 22]. Some evidence suggests that the enhancement of the acoustic startle response may be related to increased neural synchrony caused by noise exposure. Our recent study found that noise exposure to moderate levels did affect the synaptic performance of auditory neurons in the cochlear nucleus [23]. In this study, we detected increased ASR, which was in line with the increase of wave III of the ABR. These results may be related to hypersynchrony between the auditory brainstem and the midbrain, especially in the cochlear nucleus [24]. We speculate that long-term noise exposure may contribute no significant difference in the noise group before temporary impairment in the cochlear hair cells but enhanced postsynaptic properties in the auditory nerves and the cochlear nucleus [25].

The ASR pathway involves complex circuitry, and one simple model was summarized in Figure 4(a) (red line). There is still some debate about which nuclei form the primary pathway. For example, some theories suggest that sound signals transmit startle stimulus information to the caudate reticular nucleus (PnC) via DCN, VCN, cochlear root neurons, and the lateral superior olive tree, which projects it to skeletal muscle and causes the startle response [12]. We examined the nuclei in both the primary ascending pathway and the descending modulation pathway of ASR using the c-Fos, which is frequently used to indicate active neurons [17]. By comparing the number of c-Fos-positive neurons, we found significant elevations in the cochlear nucleus and the PnC which are consistent with our behavior results. Our data suggest that moderate level noise exposure contributes to an enhancement of excitation in the lower-level auditory brainstem, which may contribute to an increase of the startle response [26, 27].

Environmental noise is known to trigger stress responses in the central nervous systems [28]. During the cortical developmental period, rearing neonatal rats (P7) in 65-70 dB SPL broadband white noise can delay the segregation of cortical tonotopic maps and the frequency selectivity of the AC neurons [29]. Interestingly, a recent study found that long-term passive exposure to moderate intensity (70 dB SPL for two weeks) can reopen the critical period of cortical development of adult rats (3-4 months old) and cause expansion of the cortical area and modified prepulse inhibition (possibly related with hyperacusis) [30]. A recent study found that the recruitment of additional synaptic resources in the AC could change gap detection [31]. We speculate that long-term moderate noise exposure may synaptic expression in the central auditory system and improve auditory temporal processing.

A previous study reported that noise exposure at 110 dB SPL induced enhancement of startle response [22]. They also reported that the startle responses were increased by a background noise after they lost hearing permanently. Their results suggested that continuous background noise can prompt the startle in noise-induced sensorineural hearing loss. In our study, the background noise (Figure 3) cannot affect the amplitude of the startle response. Our results suggested that the moderate noise exposure cannot cause external hearing loss, which did not affect the startle response over background noise.

### 4.2. Low Intensity Noise Exposure Enhanced Gap Detection

A sensorimotor gating pathway mediates prepulse inhibition of the startle response. The pathway is consisted of multiple centers, including the CN, the IC, the superior colliculus, and the pedunculopontine tegmental nucleus; all nuclei deliver startle information and project to the PnC (Figure 4(a), blue line). The DCN receives both auditory input from the auditory nerve and somatosensory input, indirectly, via the axons of the granule cells of the CN [32]. The projection from the DCN and VCN to the PnC is the direct cause of ASR. The AVCN is among the most effective sites for electrically evoking startle-like responses [33]. The DCN plays a role in long-latency components of excitation of PnC neurons [34] and modulates the startle response. We found that moderate noise exposure increased c-Fos immunoreactivity in the DCN but not at higher levels of the auditory pathway. The results suggested that ASR and gap detection may be related to functional alterations in the brainstem (such as the DCN), rather than the auditory nucleus at higher levels. As increasing spontaneous rate in the DCN was also reported in noise-induced tinnitus, whether moderate-level noise exposure can lead to the same physiological modification in tinnitus is to be confirmed [26].

Gap-PPI can be used to evaluate auditory temporal acuity by detecting the onset and offset of acoustical signals [35]. As the preattentive modification of the reflex does not require auditory learning, it takes less time to evaluate auditory processing compared to methods requiring auditory training [13, 36]. We expected that gap detection would be impaired by noise exposure as hearing loss subjects typically show poor speech detection, especially in noisy environments. Surprisingly, we found that moderate-level noise exposure does not only change gap-PPI amplitude for long silent gaps (>6 ms); it also improved the detection of short gaps according to self-control in the noise group (2 and 4 ms). The results suggest that low noise exposure improved temporal processing in quiet. The result was consistent with our physiological results that moderate-level noise exposure can affect neurotransmitter release and increase the firing fidelity in the auditory brainstem [23]. Gap-PPI may be improved by synaptic recruitment in the central auditory system. These studies suggested that sound stimuli at an appropriate level can improve temporal processing perception. However, whether long-term noise treatment could also improve the signal in noise detection needs to be tested.

### 4.3. Noise Exposure Alters Long-Term Somatosensory Auditory Processing in the DCN: A Possible Basis for Hyperactivity?

The DCN is the first bimodal auditory somatosensory neuronal location, receiving auditory information from the VIIIth nerve and somatosensory input via the axons of cochlear nucleus (CN) granule cells [3]. Whether it is DCN or VCN mainly projected to PnC causing ASR is still in dispute. Aitkin et al. found evidence for a direct projection from the VCN to the PnC [37], and the anteroventral cochlear nucleus is one of the most effective areas for electrically triggering startle-like responses [38], based primarily on electrophysiological studies. DCN, on the other hand, is involved in the long-latency components of PnC neuron excitation and can retain or increase amplitude modulation coding by masking noise caused by peripheral operating range shifts [34]. In other words, long-term wound, through the auditory input pathway caused an enhancement of excitatory somatosensory inputs to the cochlear nucleus, increasing spontaneous rate in DCN^26^. There are also some researches that proved among single-unit recorded tinnitus animals, noise-induced hyperactivity was only shown in DCN [39]. Consistent with previous findings, our experiments have confirmed that long-term noise causes increased excitability of DCN after auditory peripheral injury in the morphology aspect. In addition to c-Fos staining, the wave I amplitude reduction and wave III increase is also one of the pieces of evidence.

## 5. Conclusions

In summary, we found that long-term noise exposure at low intensity could enhance ASR and gap detection, which might be indicated increased temporal processing by affecting synaptic excitability in the central auditory pathway. Our results reveal one of the perceptual consequences of increased DCN and PnC activity caused by noise exposure at low intensity.

## Data Availability

All the statistical data used to support the findings of this study are available from the corresponding author upon request.
